# Selenium and Zinc Intakes of Staple Grains and Their Correlation with Urine Selenium and Zinc in the Tibetan Rural Residents along the Yarlung Zangbo River

**DOI:** 10.3390/nu15082010

**Published:** 2023-04-21

**Authors:** Yumin Jia, Cangjue Nima, Linsheng Yang, Li Wang, Binggan Wei, Yonghua Li, Hairong Li, Yangzong Deji, Shengcheng Zhao, Min Guo, Hongqiang Gong, Chang Kong, Lijuan Gu, Zongji Gesang, Rujun Li

**Affiliations:** 1 Key Laboratory of Land Surface Pattern and Simulation, Institute of Geographical Sciences and Natural Resources Research, Chinese Academy of Sciences, Beijing 100101, China; 2 College of Resources and Environment, University of Chinese Academy of Sciences, Beijing 100049, China; 3 Tibet Center for Disease Control and Prevention, Lhasa 850030, China

**Keywords:** selenium, deficiency, zinc, grains, Tibet

## Abstract

Grains account for a large proportion of the diet of rural residents in Tibet. The lack of selenium (Se) and zinc (Zn) threatens the population’s nutrition and health. However, the intakes of selenium and zinc in grains remains unclear. To clarify the nutritional status of selenium and zinc consumed from staple grains of residents along the Yarlung Zangbo River in Tibet, 341 grain samples and 242 urine samples were collected, and 244 food frequency questionnaires were completed along the Yarlung Zangbo River in 2020–2021. The results showed that the selenium concentrations of 88.5% of self-produced tsampa and 80.8% of self-produced flour were lower than the grain selenium threshold (<25 μg·kg^−1^). The intake of selenium and zinc from staple grains (tsampa, flour, and rice) contributed 15.0% and 43.5% to the recommended nutrient intake (RNI) on average, respectively. A geographical detector model analyzed factors affecting urinary selenium and zinc levels. Selenium and zinc intakes in rice and flour, and dietary diversity score (DDS) were the main factors affecting urinary selenium and zinc (*p* < 0.01). Their interaction effects on urinary selenium and zinc were greater than those of a single factor. The staple grains of rural residents along the Yarlung Zangbo River were in a state of selenium deficiency. The zinc content of the staple grain purchased was lower than that of the main grain produced by rural residents. Changing the grain consumption pattern and adjusting the proportion of exogenous grains can improve selenium and zinc nutrition in residents.

## 1. Introduction

Selenium (Se) and zinc (Zn) are essential trace elements in human nutrition [1,2]. Selenium is a trace mineral required to maintain various functions of the body. Selenium deficiency is associated with endemic selenium deficiency, such as damage to the myocardium, leading to Keshan disease, as well as chondrocyte damage, leading to endemic osteoarthritis and Kashin–Beck disease [3,4,5]. The lack of selenium in the diet will lead to cardiovascular diseases [6], increase the incidence rate and risk of cancer [7], increase the probability of virus infection [8], reduce immunity, and cause a variety of diseases [9]. Selenium is also related to the nervous system and endocrine system [10]. Increasing dietary selenium intake is conducive to improving immunity [11], improving mental health [12], and improving male and female fertility [13]. Zinc is closely related to diet, and zinc deficiency is a global nutritional problem that affects over 30% of the world’s zinc deficient population. Zinc deficiency impairs growth and development, reduces immune function, and damages reproductive health [2,14]. It can lead to osteoporosis, dermatitis, and various diseases related to the gastrointestinal tract, liver, and kidneys [14]. Zinc supplementation can improve immunity, can be used to treat children’s acute diarrhea and respiratory diseases [15], and can reduce the risk of diabetes and cardiovascular diseases [16].

The dietary structure of residents on the Qinghai–Tibet Plateau is mainly composed of grains (44.2%), meat (10.7%), and milk (6.9%); grains account for a large proportion of the dietary structure [17]. The lack of microelements in grains significantly threatens human nutrition and health. The concentration of microelements in grains, such as selenium and zinc, is decreasing compared to what was previously noted [18,19]. Improving the mineral content of the grain diet is essential for the body’s absorption [20]. In most countries, cereals and their derivatives are the main sources of dietary selenium intake [21]. Low zinc concentrations in grains and a high intake of phytate-containing grain proteins lead to zinc deficiency [14]. Grains provide 50.1–57.2% selenium and 51.6–63.2% zinc for rural residents [22,23]. It is of great significance to study the concentration of selenium and zinc in grains for evaluating the residents’ selenium and zinc intakes.

In most parts of China, the selenium concentration in soil, water, plants, the human diet, and human hair is very low. Approximately 51% of the soil in China is deficient in selenium [24]. Many local soils in Tibet are low in selenium, with a mean soil concentration lower than the Chinese average [25]. The low selenium environment affects the selenium content of local crops [26]. With the development of the economy and improvement in living conditions, exogenous foods have increased, and dietary foods have become more diversified. However, locally produced food, especially special grain tsampa, which is made of roasted highland barley grains, still forms a part of local rural residents’ diets. Residents cannot completely break away from dependence on locally produced food, and due to uneven regional development, this dependence also forms regional differences. There is still a large gap between the daily selenium intake of rural residents in the low-selenium zone and their physiological need for selenium [27]. However, there is no systematic study on whether dependence on self-produced grain creates a hidden danger for low selenium levels in rural residents.

Grains, meat, eggs, and milk are the four major food sources of selenium and zinc for rural residents in Tibet, among which grains account for the largest proportion, contributing 54.1% to the total dietary selenium intake and 36.0% to the total dietary zinc intake [27]. The study areas of Xigaze and Lhoka are located in southwest Tibet and are important grain production areas. The total planting area of crops in Xigaze and Lhoka is 127.2 thousand hectares, accounting for 47.7% of the Tibet Autonomous Region [28]. There has been no large-scale investigation into the selenium and zinc content of the local grain, especially the special grain tsampa, which is made from fried highland barley grains. Therefore, it is necessary to study selenium and zinc in grains, especially after processing, as grains also contain a large number of anti-nutritional compounds, such as phytate (inositol phosphate compound), which inhibits the absorption of zinc in human intestines [29]. The lack of antinutrients for local staple foods leads to a lack of quantitative data on zinc intake. Consequently, it is important to determine the selenium and zinc content in grains and the grain intake to assess this nutritional status in local residents.

This study selected Xigaze and Lhoka as representative sample areas and, through the analysis of the staple food and urine samples, aimed to explore (1) the local residents’ selenium status, and the selenium and zinc contents of grains consumed; (2) the quantity of selenium and zinc obtained by local residents from grain and the dependency on self-produced grains. We also used geographical detectors to explore (3) the extent to which the selenium and zinc intakes of staple grains affect human selenium and zinc levels, and whether the impact on human selenium and zinc levels is higher in self-produced or purchased staple grains. This study provides a scientific basis for evaluating local grains’ selenium and zinc contents, guiding the rational distribution of grain planting, and improving selenium and zinc nutrition among local residents.

## 2. Materials and Methods

### 2.1. Study Area and Sampling Points

The study area is located in southwest Tibet, China, covering the cities of Xigaze and Lhoka, through which the Yarlung Zangbo River, the largest river in Tibet, flows. The study was approved by the Tibet Center for Disease Control and Prevention and was jointly carried out on the basis of signing a cooperation agreement (20200731). The procedure of this study was in line with the ethical standards of the Institutional Research Committee, as well as the guidelines of the Declaration of Helsinki. All subjects gave their informed consent for inclusion before they participated in the study. Our study population consisted mainly of residents over 18 years living in rural areas. In this study, with the farmers’ knowledge and consent, we collected daily staple food and urine samples from farmers’ homes and randomly selected permanent residents for a questionnaire survey. Grain and urine samples from farmers’ homes and the food frequency questionnaires were completed in 2020–2021 on both sides of the Yarlung Zangbo River.

### 2.2. Sample Collection

**Figure 1:**
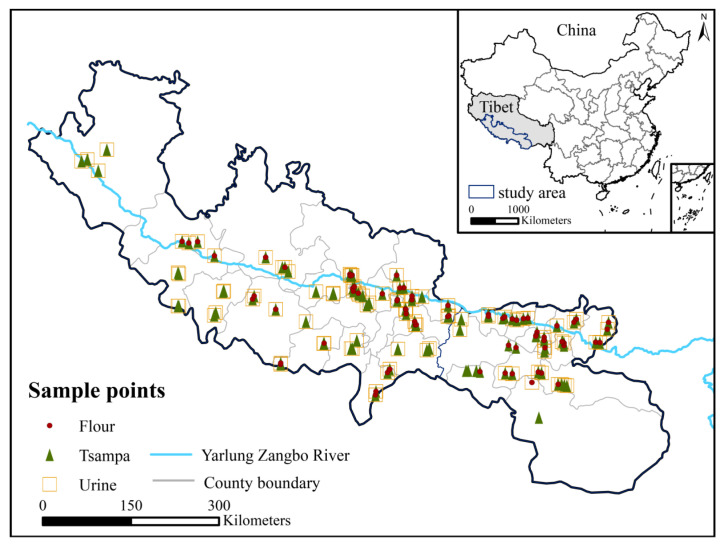
Sampling locations.

We selected two townships for each county, two villages for each township, and randomly selected two households for sample collection and a questionnaire survey in thirty counties on the north and south sides of the Yarlung Zangbo River (Figure 1). We collected grain samples (tsampa, flour, and rice) from residents’ homes and recorded the sample information. At the same time, we indicated the source of the sampled grain samples, which were self-produced or purchased by farmers at the market. Finally, the samples were sealed and stored. After obtaining the farmers’ consent, we collected random urine samples, selected the middle period of the entire random urine process, and recorded the name, age, gender, and sampling time of the farmers. The collected urine was transferred to 50 mL polypropylene centrifuge tubes, labeled, and numbered, and then transferred to 2 mL, 5 mL, and 10 mL freezer tubes for sample labeling. The urine samples were stored in a 4 °C on-board refrigerator sealed on site, transferred to the local Centers for Disease Control and Prevention, transported to the laboratory, immediately stored in a −20 °C refrigerator, and analyzed within 3 months. The GPS instrument was used to record the geographic location information of sampling points.

A food frequency questionnaire was used to investigate the diets of the farmers. The questionnaire consisted of basic information on daily food consumption. The basic information included age and gender; daily food consumption included the frequency and sources of different foods that the residents consumed. A total of 341 grain samples, 242 urine samples, and 244 questionnaires were collected.

### 2.3. Chemical Analysis

The grain samples were weighed to approximately 0.5000 g and digested with 3 mL of HNO_3_ by electrothermal heating. After digestion, the volume was adjusted to 20 mL. Grain Se was determined using inductively coupled plasma mass spectrometry (ICP–MS) (Agilent Technologies 8900, Santa Clara, CA, USA), and the collision pool was added to the selenium measurement process. Zn content was determined using an inductively coupled plasma optical emission spectrometer (ICP–OES) (Avio 500, PerkinElmer, Waltham, MA, USA).

The urine samples were thawed at room temperature and shaken thoroughly. One milliliter of raw urine was added to 1 mL HNO_3_ overnight. After digestion, the liquid was cleared without precipitation, and the volume was fixed at 10 mL. Urine Se was determined by ICP–MS (Agilent Technologies 8900, Santa Clara, CA, USA), and Zn was determined by ICP–OES (Avio 500, PerkinElmer, Waltham, MA, USA). The collision pool was added for the determination of selenium.

The phytic acid content in the grains was determined using a Microplate Reader (M2e, Molecular Devices, San Jose, CA, USA) and a Phytic Acid Assay Kit (Soponin). The determination principle is that the sulfosalicylic acid–ferric chloride solution is purplish-red and has a maximum absorption value of 500 nm. In the pH 6.0 to 6.5 environment, phytic acid and iron ions were combined to lighten the solution color and determine the reduction in absorbance to detect phytic acid content.

The detection limits of Se and Zn were 0.01 μg·L^−1^ and 1 μg·L^−1^ in the obtained solutions of staple samples. The range of the correlation coefficients of the calibration regression curves for the standard solution’s two elements was 0.9995–0.9999. The relative standard deviation in duplicate samples ranged from 2.5 to 4.6%.

National standard material GBW100495 (wheat flour, obtained from the National Institute of Metrology of China in Beijing) was used for the quality control of grain samples; the relative error of selenium measurement in the standard sample was 1–5%, and the relative error of zinc measurement was 2–5%. Parallel samples were set for each sample type, and 10% were randomly selected for repeated determination. The relative deviation range of parallel double samples was 5–8% for selenium, 2–7% for zinc, and 1–4% for phytic acid, which met the test accuracy requirements. The blank samples were analyzed and measured similarly to the experimental samples.

### 2.4. Calculation and Statistical Analysis

#### 2.4.1. Grain Se and Zn Intakes

We calculated the selenium and zinc intakes from staple grains using the following formulas:

(1) $${DGI}_{Se}=\sum_{i=1}^{3}C_{i}\cdot A\cdot F_{i}$$

(2) $${DGI}_{Zn}=\sum_{j=1}^{3}C_{j}\cdot A\cdot F_{j}$$

(3) $${F_{j}=F}_{i}=\frac{f_{i}}{\sum_{i=1}^{3}f_{i}}$$

where ${DGI}_{Se}$ is the daily grain Se intake (μg∙d^−1^), *i* = 1, 2, 3 correspond to tsampa, flour, and rice, respectively, and *C_i_* is the Se concentration in the grain (μg∙kg^−1^). $F_{i}$ is the proportion of the consumption of each grain to the total grains. $f_{i}$ is the daily consumption frequency of grains (times/day). ${DGI}_{Zn}$ is the daily grain Zn intake (μg∙d^−1^), *j* = 1, 2, 3 correspond to tsampa, flour, and rice, $C_{j}$ is the Zn concentration in the grain (mg∙kg^−1^). A = 0.4376 kg∙d^−1^ is the daily grain intake, referring to the data from *The Exposure Factors Handbook of Chinese Population (Adults)* [30].

#### 2.4.2. Estimation of Phytate: Zinc Molar Ratio

We considered the effect of phytate in grains on zinc absorption in the human body. The estimation of the molar ratio of phytate to zinc was based on the following formula:

(4) $$R_{PZ}=\frac{\frac{P_{t}}{M_{P}}}{\frac{{Zn}_{i}}{M_{Zn}}}$$

where $R_{PZ}$ is the phytate–zinc molar ratio, $P_{t}$ is the phytate content of the grain (mg∙kg^−1^), and ${Zn}_{i}$ is the Zn content of the grain (mg∙kg^−1^). $M_{P}$ is 660.04 molar weight of phytate; $M_{Zn}$ is 65.38 molar weight of Zn.

#### 2.4.3. Estimation of Absorbable Zinc

According to previous studies, the model proposed by Miller is relatively reliable for calculating the zinc absorption fraction [31].

(5) $$FAZ=\frac{0.5}{TDZ}\cdot \left\{A_{MAX}+TDZ+K_{R}\cdot \left(1+\frac{R_{PZ}TDZ}{K_{P}}\right)-\sqrt{{\left(A_{MAX}+TDZ+K_{R}\cdot \left(1+\frac{R_{PZ}TDZ}{K_{P}}\right)\right)}^{2}-4\cdot A_{MAX}\cdot TDZ}\right\}$$

(6) $$TDZ=\frac{{DGI}_{Zn}}{M_{Zn}}$$

(7) $${DGI}_{Zn-actual}=\sum_{j=1}^{3}C_{j}\cdot A\cdot F_{j}\cdot {FAZ}_{j}$$

The model has three parameters: $A_{MAX}$, $K_{R}$, and $K_{P}$. *A*_MAX_ is the maximal absorption of zinc; $K_{P}$ and $K_{R}$ are the equilibrium dissociation constants of zinc-phytate and zinc-receptor binding, respectively [31]. FAZ is the fractional absorption of zinc, TDZ is the dietary zinc (mmol), and $R_{PZ}$ is the phytate–zinc molar ratio. The fractional absorption of zinc was calculated using the latest model parameters [32]. The actual daily grain zinc intake was multiplied by FAZ.

#### 2.4.4. The Contribution to the Recommended Nutrient Intake

The recommended nutrient intake (*RNI*) refers to the intake level of a certain nutrient that can meet the needs of most individuals (97–98%) in a specific gender, age, and physiological condition group. Long-term intake of nutrients at the *RNI* level can meet the body’s needs. This study calculated the contribution of selenium and zinc intakes in grains to the recommended nutrient intake. The study subjects were all rural residents over 18 years of age, and the recommended selenium intake was 60 μg∙d^−1^. The recommended nutrient intake of zinc varies by sex and is 12.5 mg∙d^−1^ for men and 7.5 mg∙d^−1^ for women [33].

(8) $$\frac{Se\;intake}{RNI}=\frac{{DGI}_{Se}}{{RNI}_{Se}}\times 100\%$$

(9) $$\frac{Zn\;intake}{RNI}=\frac{{DGI}_{Zn-actual}}{{RNI}_{Zn}}\times 100\%$$

#### 2.4.5. Dietary Diversity Score

The dietary diversity score (DDS) indicates dietary diversity based on food category counts [34]. Based on the characteristics of the dietary habits of the local residents, the food, except main grains, was divided into 10 food types, including corn, potato and products, dairy products, beans and products, meat, poultry, eggs, fruits, vegetables, and edible oil. DDS calculates the frequency of consumption of various types of food by residents in the previous month. Each type of food consumed was counted as 1 point. Regardless of the frequency and quantity of consumption, the highest score was 13 points.

#### 2.4.6. Geographical Detector

Geodetectors are a group of statistical methods used for detecting spatial differentiation and revealing the driving forces behind them. The geographic detector consists of four detectors: factor, interaction, ecological, and risk detectors [35]. The effects of grain selenium and zinc intakes on urinary selenium and zinc levels were studied using factor and interaction detectors.

#### 2.4.7. Statistical Analysis and Mapping

The concentration statistics, group comparison, and mapping of the main grains were completed using Origin 2021 (OriginLab, Northampton, MA, USA, Windows version 2021). The Mann–Whitney U test was used to compare two independent samples. The Kruskal–Wallis H test was used to compare multiple independent samples, and the non-normal distribution data were used for the non-parametric Spearman test. *p* values < 0.05 (two–tailed) were considered statistically significant. The distribution of sampling points and spatial distribution of Se and Zn concentrations in staple grains were determined using ArcMap (version 10.5, ESRI Inc., Redlands, CA, USA). The free geodetector software used in this study is downloaded from http://geodetector.cn/.

**Figure 2:**
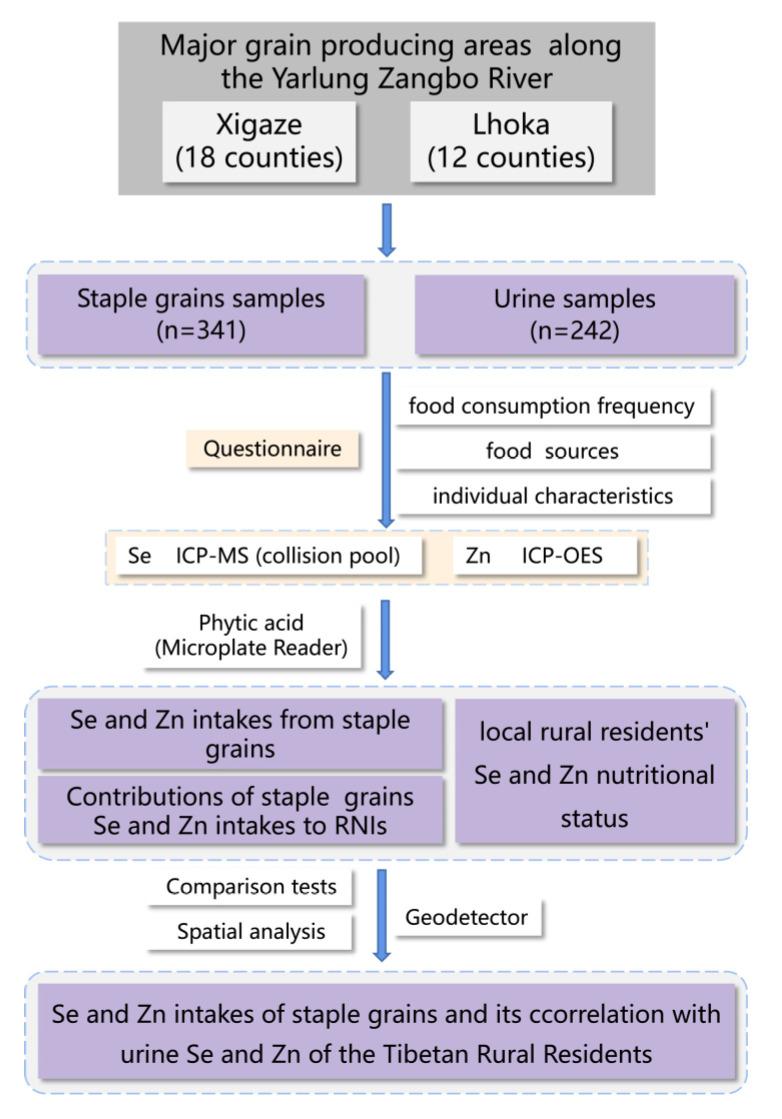
Study method.

The study procedure is shown in Figure 2.

## 3. Results

### 3.1. Concentrations of Se and Zn in Self-Produced Staple Grains

**Figure 3:**
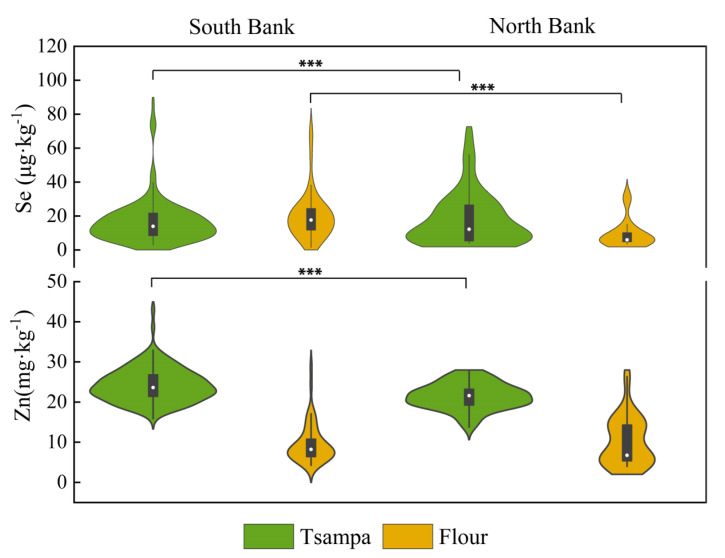
Se and Zn concentrations of staple grains on both banks of Yarlung Zangbo River (Boxes within the violins show the interquartile range, and medians are marked as white dots; whiskers indicate 1.5 times of interquartile range; *** indicates that the *p* < 0.01).

The analysis of self-produced household staple grains showed that the concentrations of Se and Zn were significantly different on both banks of the Yarlung Zangbo River (Figure 3). Se concentration in tsampa on the south bank of the Yarlung Zangbo River ranged from 2.85 μg·kg^−1^ to 86.58 μg·kg^−1^ (mean 16.79 ± 13.46 μg·kg^−1^), significantly higher than that in the north bank (13.50 ± 13.13 μg·kg^−1^; range 1.71–56.45 μg·kg^−1^) (*p* < 0.01). For Se in flour, the south bank (average 20.06 ± 14.13 μg·kg^−1^; range 1.48–71.47 μg·kg^−1^) was also significantly higher than the north bank, and the average value was even more than three times that of the north bank (average 6.41 ± 7.00 μg·kg^−1^) (*p* < 0.01). The Zn concentration of tsampa on the south bank ranged from 15.86 to 43.06 mg·kg^−1^, and the average value was 24.21 ± 4.25 mg·kg^−1^. It was also significantly higher than the north (average 21.45 ± 3.22 mg·kg^−1^, range 13.73–27.67 mg·kg^−1^) (*p* < 0.05). However, there was no significant difference in the zinc content of the flours between the two sides.

Tsampa was a good potential source of Zn, with an average Zn concentration (23.57 ± 4.19 mg·kg^−1^; range 13.73–43.06 mg·kg^−1^), exceeding more than twice than that of self-produced flour (average 9.78 ± 5.19 mg·kg^−1^). People who rely on tsampa as their staple food may have a higher Zn intake. According to the questionnaire survey, 99.6% or almost all residents ate tsampa daily. The proportions of residents who consumed flour and rice daily were 53.7% and 72.5%, respectively (Table A1). In addition, more than 50% of the total residents and more than 90% of the residents in the survey bought flour and rice. Tibetan traditional food tsampa is an important prominent grain for the locals, but the staple grain purchased also occupies a certain proportion.

The selenium content of the purchased staple grain was significantly higher than that of the flour (*p* < 0.01), whereas the zinc content was lower than that of the self-produced main grain. The average selenium content of purchased flour was 25.22 ± 10.50 μg·kg^−1^, which was significantly higher than that of self-produced flour (average 16.39 ± 13.96 μg·kg^−1^) (*p* < 0.01). The mean zinc content of purchased flour was 6.81 ± 3.44 mg·kg^−1^, which was significantly lower than that of self-produced flour (average 9.78 ± 5.19 mg·kg^−1^) (*p* < 0.01). The average selenium content of the purchased rice was 34.12 ± 18.13 μg·kg^−1^, significantly higher than that of the self-produced flour and tsampa (*p* < 0.01). The average zinc content of the outsourced rice was 12.70 ± 1.38 mg·kg^−1^, which was also significantly lower than tsampa (*p* < 0.01).

Therefore, locally produced grains were not an adequate source of selenium for residents and purchased main grains were a good source of selenium. Local grain tsampa was a good zinc source for residents, much higher than the zinc content of outsourced flour and rice. The role of self-produced foods should also be considered when considering zinc nutrition.

### 3.2. Geospatial Variation of Se and Zn in Staple Grains

**Figure 4:**
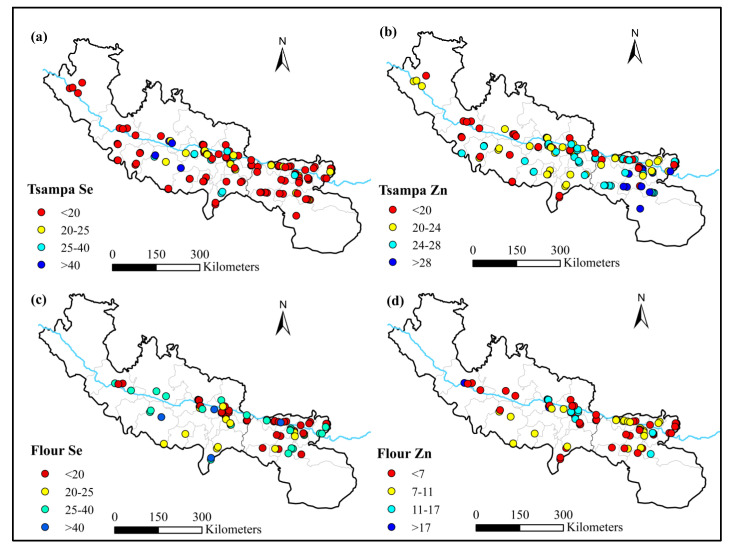
Spatial distribution of Se (μg·kg^−1^) and Zn (mg·kg^−1^) concentrations of staple grains. (**a**) Spatial distribution of Se (μg·kg^−1^) concentration of tsampa; (**b**) spatial distribution of Zn (mg·kg^−1^) concentration of tsampa; (**c**) spatial distribution of Se (μg·kg^−1^) concentration of flour; (**d**) spatial distribution of Zn (mg·kg^−1^) concentration of flour.

Based on the concentrations of selenium and zinc in tsampa and flour at the sampling points, a spatial concentration distribution map was drawn. The color of selenium and zinc concentrations in tsampa and flour were indicated as large (red) and small (blue) (Figure 4). Tsampa and flour were selected because their sampling points had good spatial coverage. It can be seen from the results that the tsampa and flour consumed by the residents were in a state of selenium deficiency. Almost all samples were selenium deficient (<40 μg·kg^−1^), and 87.5% of tsampa samples were severely selenium deficient, below the selenium deficiency threshold of grain (25 μg·kg^−1^) [36]. On the south bank of the Yarlung Zangbo River, the distribution of relatively high selenium concentrations was higher than that on the north bank. The middle part of the Yarlung Zangbo River was relatively high. The lower tsampa selenium concentration was observed southeast of the study area. The selenium content of flour was higher than that of tsampa. However, it was selenium deficient, and the proportion of severe selenium deficiency was 71.6%. Selenium in the flour was relatively high in the middle part of the Yarlung Zangbo River. The Zn concentration of tsampa in the southeast was higher than in the southwest. The Zn concentration of flour was much lower than that of tsampa in the lower reaches of the Yarlung Zangbo River.

### 3.3. Staple Grains Contribution to Se and Zn Intakes

**Figure 5:**
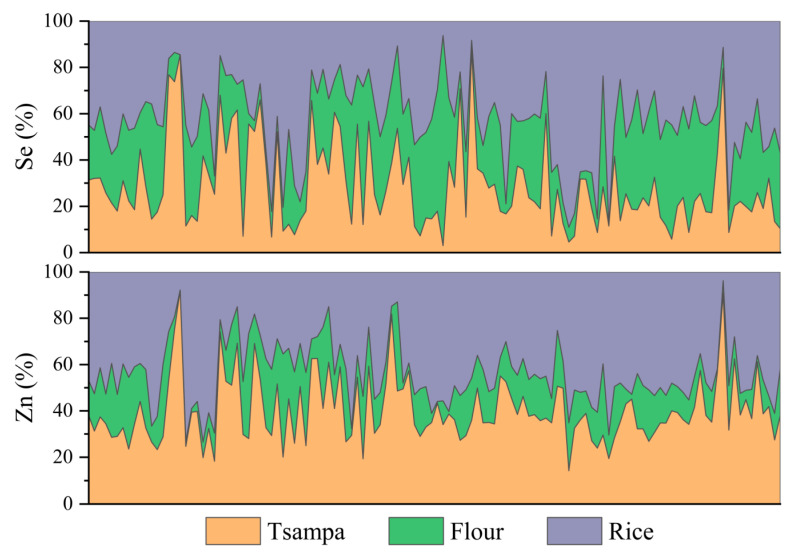
Staple grains contribution to Se and Zn intakes.

The total intakes of selenium and zinc in the staple grains and the consumption frequency of the main grains in the questionnaire, and the total intakes of selenium and zinc in the staple grains were calculated using formulas (1) and (7). The contribution of each main grain to the total intakes of selenium and zinc was calculated (Figure 5). The average contributions of rice to total intakes of selenium and zinc were 43.3% and 44.1%, respectively. It can be seen that rice, which was all purchased, was a valuable source of selenium and zinc in comparison to other grains. For tsampa and flour, the average contributions to the total intake of selenium were 29.2% and 27.5%, respectively, much lower than that of rice, as the selenium content of tsampa and flour was much lower. The average contribution to the total zinc intake in tsampa (40.4%) was significantly higher than that in flour (15.5%) (*p* < 0.01). This is attributed to the much higher zinc content of tsampa than flour. However, it was slightly lower than rice because the FAZ of tsampa was 36.6%, lower than that of rice (62.6%), and the inhibitory effect of phytic acid on zinc absorption was stronger in tsampa.

**Figure 6:**
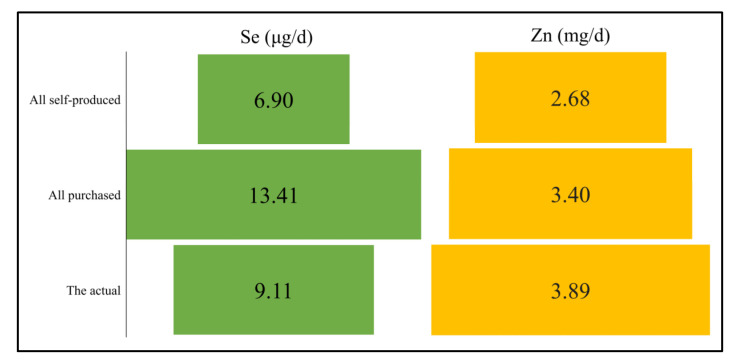
Se and Zn intakes of three assumptions.

The extent of the contribution of outsourcing staple grains to selenium and zinc intakes was further assessed. It is assumed that all local people eat self-produced staple food, that is, tsampa and self-produced flour, and that all local people eat purchased staple food, rice, and flour. The intakes of selenium and zinc corresponding to these two conditions are shown in Figure 6. When all the purchased main grains were consumed, the selenium intake was 13.41 μg·d^−1^, and that was thus 6.90 μg·d^−1^, almost twice that of the latter. Zinc intake was also higher in all purchased staple grains. However, the selenium intake of the staple grains in actual situation was 9.11μg·d^−1^, which is higher than all self-produced situation (6.90 μg·d^−1^) and lower than all purchased situation (13.41 μg·d^−1^). The actual zinc intake was 3.89 mg·d^−1^, exceeding the all produced (2.68 mg·d^−1^) or outsourced zinc (3.40 mg·d^−1^). Rice and tsampa had notably higher concentrations of zinc. In comparison to the theoretical situation where all grains are produced, rice purchased as a staple food contributed more than 40% to the overall zinc intake in the actual situation. Meanwhile, in comparison to the situation where all grains are purchased, tsampa produced locally contributed over 40% of the total zinc intake in the actual situation. Therefore, the actual situation shows a higher zinc intake than that of all self-produced or purchased staple grains.

**Figure 7:**
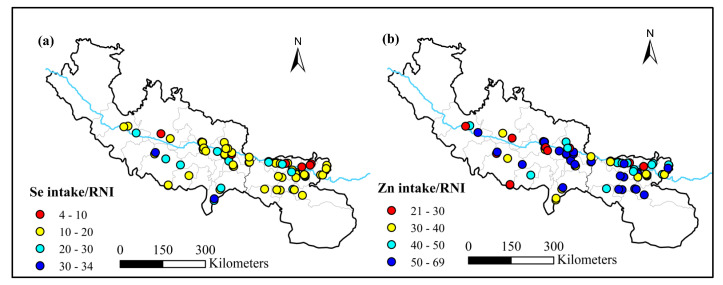
Contributions of Se and Zn intakes to RNIs (%). (**a**) Contributions of Se intake to RNI (%); (**b**) contribution of Zn intake to RNI (%).

The contribution of selenium to the recommended nutrient intake ranged from 4% to 34% (Figure 7). Higher values were more distributed on the south bank of the Yarlung Zangbo River, and the south bank (average 16.9%) was significantly higher than that of the north bank (average 10.9%) (*p* < 0.01). The contribution of zinc to recommended nutrient intake ranged from 21% to 69%. The south bank (average 45.2%) was also significantly higher than the north bank (average 39.5%) (*p* < 0.01). Therefore, south bank residents obtained higher levels of selenium and zinc from grains than from the north shore.

### 3.4. The Relationship between the Concentration of Selenium and Zinc in Urine and the Intakes of Selenium and Zinc in Staple Grains

#### 3.4.1. Spatial Distribution of Urine Sample Selenium and Zinc

**Figure 8:**
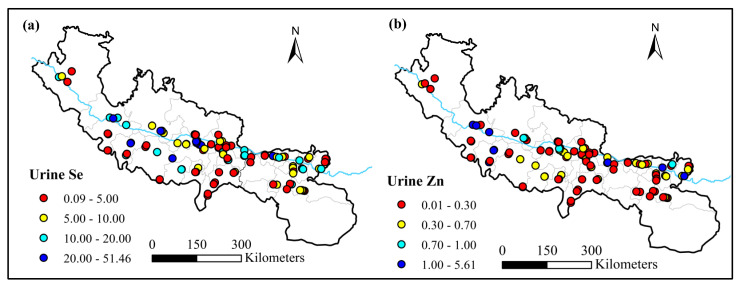
Distribution of Se (μg·L^−1^) and Zn (mg·L^−1^) concentration in urine samples. (**a**) Urinary Se (μg·L^−1^); (**b**) urinary Zn (mg·L^−1^).

The urine selenium content of rural residents in the sampling area ranged from 0.09 to 51.46 μg·L^−1^, with a mean value of 6.89 ± 7.24 μg·L^−1^. The urine zinc range was 0.01–5.61 mg·L^−1^, and the mean value was 0.33 ± 0.47 mg·L^−1^ (Figure 8). In the southeast of the study area and the south bank of the Yarlung Zangbo River, the urinary selenium value was low, and the low distribution of urinary zinc was also concentrated in the southeast of the study area.

#### 3.4.2. Analysis of Influencing Factors Based on Geographical Detector Model

We used a factor detector to determine the effect of multiple factors, such as the intakes of selenium and zinc in the main grains, on the content of selenium and zinc in urine (Table 1). The q-statistic of the factor detector results indicates the explanatory power of a factor for spatial differentiation. The range of q-statistic is [0, 1]. The larger the q-statistic, the stronger the explanatory power of the independent variable to the dependent variable [37]. For urine, selenium and zinc in flour and rice had the greatest impact on urine selenium. Next, there was DDS. This means that increasing the purchased food and food diversity will help to improve the selenium concentration in the urine. Rice had the greatest impact on the concentration of zinc in urine, followed by DDS. Selenium and zinc in self-produced flour also significantly impacted urine selenium, indicating that outsourced food and self-produced food can promote the concentration of zinc in urine. However, selenium intake (*p* > 0.05) did not significantly affect urine zinc levels.

**Figure 9:**
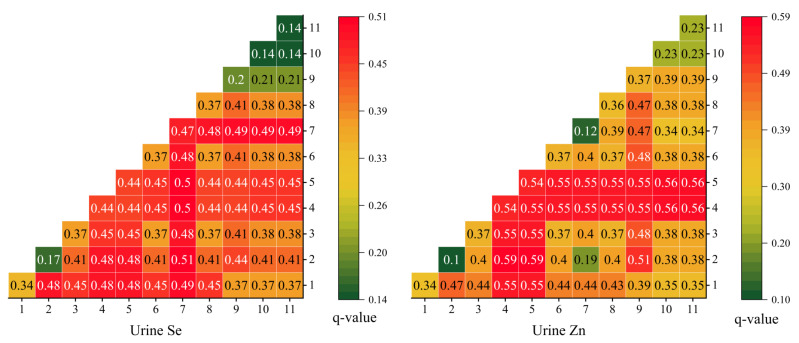
Interactive detector results (Numbers 1–11 correspond to the 11 factors listed in Table 1).

The interaction detection analysis of 12 influencing factors shows that the interaction between multiple factors presents a double factor enhancement or nonlinear enhancement; that is, the content of selenium and zinc in urine is the result of the interaction of multiple influencing factors (Figure 9). The higher the interaction q- statistic, the greater is the impact of the interaction between the two corresponding factors on urine selenium or zinc. For urinary selenium, the intake of selenium in the main grains and the interaction q value of selenium and zinc in DDS, rice, and flour were both higher, showing a double-factor enhancement. The interaction q value of flour selenium and other factors was higher than 0.48, indicating a double-factor enhancement. This shows that the selenium content in urine is affected by the selenium intake of the main grains and other factors. The main grains with external sources, such as flour and rice, affected the selenium content in urine by affecting the intake of selenium, while the self-produced main grains had less impact. For urine zinc, the interaction of Se and Zn in rice with other factors showed a double-factor enhancement, with an interaction value of more than 0.54, slightly higher than the explanatory power of a single factor of rice, indicating that the interaction had a greater impact on urinary zinc. Still, the content of Se and Zn in rice was the main influencing factor.

## 4. Discussion

In our study, the average selenium concentration of tsampa produced on the south bank of Yarlung Zangbo River was 16.79 ± 13.46 μg·kg^−1^, which was consistent with the average tsampa grain (0.017 ± 0.003 mg·kg^−1^) of Zhou et al. [38] along the Yarlung Zangbo River, and slightly lower than the selenium concentration of tsampa collected on the south bank of Guo et al. [39] (16.98 ± 7.93 μg·kg^−1^). The average selenium concentration of self-produced tsampa on the north bank was 13.50 ± 13.13 μg·kg^−1^, which was significantly lower than that on the south bank but higher than that in the Yarlung Zangbo River collected by Guo et al. (9.29 ± 8.58 μg·kg^−1^) [39]. It was also higher than the mean value of selenium concentration of tsampa grain in Songpan County (9.0 ± 8.8 μg·kg^−1^) of Wang et al. [40], and the mean value of total selenium in tsampa grain in the Kashin–Beck disease area of Tibet (10.51 ± 5.18 μg·kg^−1^) of Zhang et al. [41]. The selenium concentration of tsampa and flour on the south bank of the Yarlung Zangbo River was significantly higher than that on the north bank, consistent with the research conclusions of Guo et al. [39] for the Yarlung Zangbo River. This is because the selenium concentration in the soil on the north bank is significantly lower than that on the south bank, and the low selenium content in tillage soil leads to low selenium content in grains [38]. Overall, the selenium concentrations in 80.8% flour and 88.5% tsampa were lower than the threshold values for Se deficiency in grains (25 μg·kg^−1^). Most of the self-produced grains were in a state of selenium deficiency, and the proportion of selenium deficiency was relatively high in both the northern and southern banks, among which the proportion of flour in the north bank was as high as 94.4%. This shows that locally produced grains are not an effective source of Se for residents.

The average zinc concentration of self-produced tsampa on the south bank was 24.21 mg·kg^−1^, and that on the north bank was 21.45 mg·kg^−1^. These values were slightly lower than tsampa in non-Kashin–Beck-disease areas on the north bank of the Yarlung Zangbo River [42] (27.11 ± 3.44 mg·kg^−1^). They were not significantly different from the zinc concentration of Zha et al. [43] in the Kashin–Beck disease areas of Tibet (23.58 ± 3.10 mg·kg^−1^). The average concentration of zinc in self-produced flour was 9.78 mg·kg^−1^, ranging from 3.94–28.96 mg·kg^−1^, which was lower than that of Guo et al. [42] on both sides of the Yarlung Zangbo River (10.97 ± 4.67 mg·kg^−1^ in non-diseased areas on the north bank; 12.79 ± 3.34 mg·kg^−1^ in non-diseased regions on the south bank). In addition, the concentration of zinc in flour is far lower than the global average level of flour (mean 27.3 mg·kg^−1^, range 20.4–30.5 mg·kg^−1^), and the average value of zinc content in Chinese flour grains (23.3 mg·kg^−1^, 10.1–49.7 mg·kg^−1^) [44]. Moreover, a mature grain’s critical zinc concentration range is 20–24 mg·kg^−1^, whereas the zinc concentrations of 57.7% of self-produced tsampa and 95.6% of self-produced flour were lower than 24 mg·kg^−1^ in our study [45]. This shows that zinc deficiency exists in grains and cannot be ignored. Zhou et al. [27] investigated 14 agricultural counties along the Yarlung Zangbo River and found that Tibetan residents lacked zinc and selenium intake. The effects of grains on Se and Zn nutrition cannot be ignored.

In this study, the average contribution of selenium intake to RNI was 15.2%, which was lower than the contribution of grain to selenium intake in rural residents in China (50.1%) [33]. The higher values were more distributed on the south bank of the Yarlung Zangbo River, possibly due to the higher selenium concentration of the self-produced staple food on the south bank than on the north bank. In other studies along the Yarlung Zangbo River, based on the food composition table, local residents’ daily dietary selenium intake was less than 45% of China’s RNI [27]. Therefore, the main grain selenium is still an important source of selenium for local residents. Purchased main grain (rice) contributed the most to selenium intake (43.3%), followed by tsampa and flour.

The zinc intake obtained from the main grains accounted for 43.5% of China’s RNI on average, which was lower than the contribution of grain to the zinc intake of rural residents in China (56.3%) [33]. Considering the inhibitory effect of phytic acid on zinc absorption, rice’s contribution rate to the main grains’ zinc intake was 44.6%, and that of tsampa to the zinc intake of the main grains was 40.4%. In other words, purchased rice and tsampa were the main sources of Zn obtained by local residents from the main grains. These results are consistent with those of the factor and interaction detectors. Imported staple food affects selenium and zinc in the human body.

Therefore, locally produced grains are not an effective source of selenium for residents and purchased main grains are a good source of selenium. Selenium intake can be increased by increasing the proportion of imported staple foods. In addition, some selenium crop supplements to improve selenium bioavailability are considered feasible methods [46]. Local grain tsampa is a good zinc source for residents, much higher than the zinc content of outsourced flour and rice. The role of self-produced grains should also be considered when considering zinc nutrition. Furthermore, selenium and zinc intakes can be improved by increasing the food diversity and increasing the consumption of foods with high selenium content or zinc content, such as meat, seafood, eggs, and vegetables.

Urine is considered a good indicator of selenium levels in the body, and zinc in urine also changes with zinc intake [47,48,49]. The mean concentration of urinary selenium in the study area was lower than in other parts of China. For example, urine selenium was lower than that of rural residents in Hainan, China (median 36.5 μg·L^−1^) [50], and urine selenium of rural residents in Lhasa (male 13.27 ± 12.88 μg·L^−1^; female 11.66 ± 9.94 μg·L^−1^) [51], which was also lower than the average of Chinese adult males (7.9 μg·L^−1^) [52]. Compared with other regions in China, the average concentration of urine zinc was lower than that of rural residents along the Yangtze River (male 0.46 mg·L^−1^; female 0.52 mg·L^−1^) [53] and Chinese adult males (0.38 mg·L^−1^) [52], but higher than Wuhan adults (0.27 mg·L^−1^) [54] and Lhasa rural residents (male 0.19 mg·L^−1^; female 0.18 mg·L^−1^) [51]. Studies have found a positive Zn–Se correlation between diet and urine, and Zn–Se has a highly significant correlation in urine [52]. In this study, urine zinc and selenium resulted from combined factors, including selenium and zinc intakes in the main grains and selenium and zinc in urine. There was also a high correlation between selenium and zinc levels in the urine samples (r = 0.83, *p* < 0.01).

This study analyzed the selenium and zinc content of staple foods consumed by rural residents in Xigaze and Lhoka, covering all the counties in these two cities, and clarified the spatial distribution which has practical significance for assessing dietary nutrition and improving crop planting structure. The phytic acid in grains inhibits the absorption of zinc in the human body. This study has considered this phenomenon and calculated the zinc absorption fraction in combination with phytic acid, which provided more reference value for the zinc intake in the staple grains. Furthermore, we made the analysis of the relationship between the concentration of selenium and zinc in urine and the intakes of selenium and zinc from staple grains, which will help us to understand the nutritional status of selenium and zinc in rural Tibet residents, and also provide reference for the formulation and implementation of dietary nutrition intervention policies for rural Tibet residents. However, our study did not investigate the complete transmission chain of soil–plant–dietary intake–human body. Moreover, only the relationship between selenium and zinc intakes from staple grains and selenium and zinc in urine samples has been explored, without further study of causality. The study had a small sample size, which may have partly affected the accuracy of the results, and further research is needed on the influencing factors of selenium and zinc in urine samples. In the future, we will conduct in-depth research into these aspects.

## 5. Conclusions

In summary, the self-produced grain in the Yarlung Zangbo River basin was in a low selenium state, and the selenium content of grains on the south bank of the Yarlung Zangbo River was higher than that on the north bank. The zinc content of the self-produced grains was higher than that of the purchased grains; however, the utilization efficiency of zinc was low. Purchased staple foods had a more obvious effect on increasing selenium intake. The contribution of selenium and zinc intakes in staple grains to RNI was lower than the contribution of grain to selenium and zinc intakes in rural residents in China. The selenium and zinc contents in urine are the result of multiple factors. The selenium and zinc contents in the purchased main grains were the main factors affecting the selenium and zinc content in the urine. Exogenous food reduces the dependence of local residents on a low-selenium environment. It is important to change the consumption structure of staple grains, give full play to the market, and further refine the collocation of grain planting and outsourcing in the region for the balance of dietary selenium and zinc nutrition.

## Figures and Tables

**Table 1:** q-statistics of different factors on urine Se and Zn.

		Urine Se		Urine Zn	
No.	Factor	q Statistic (%)	*p*-Value	q Statistic (%)	*p*-Value
1	Total Zn intake	34.46	<0.01	33.66	<0.01
2	Total Se intake	16.77	0.013	10.42	0.933
3	DDS *	37.15	<0.01	36.83	<0.01
4	Rice Zn	43.81	<0.01	54.41	<0.01
5	Rice Se	43.81	<0.01	54.41	<0.01
6	Flour Zn	37.15	<0.01	36.83	<0.01
7	Flour Se	47.14	<0.01	12.44	<0.01
8	Self-produced flour Zn	19.67	<0.01	37.24	<0.01
9	Self-produced flour Se	36.56	<0.01	35.84	<0.01
10	Tsampa Zn	13.79	<0.01	22.82	<0.01
11	Tsampa Se	13.79	<0.01	22.82	<0.01

* DDS indicates dietary diversity score.

## Data Availability

The data presented in this study are available on request from the corresponding author and with permission of the Tibet Center for Disease Control and Prevention. The data are not publicly available due to confidentiality requirements.

## Appendix A

**Table A1:** Frequency of staple grain consumption in the questionnaire.

Frequency	Tsampa (%)	Flour (%)	Rice (%)
Once a day	67.21	14.34	14.34
Twice a day	32.38	39.34	58.20
1–3 times a week	0.41	18.85	12.70
4–6 times a week	0	24.59	13.11
1–2 times a month	0	1.64	1.64
No/occasionally	0	1.23	0
